# Differentiation between bipolar disorder and major depressive disorder in adolescents: from clinical to biological biomarkers

**DOI:** 10.3389/fnhum.2023.1192544

**Published:** 2023-09-15

**Authors:** Ruilan Yang, Yanmeng Zhao, Zewen Tan, Juan Lai, Jianshan Chen, Xiaofei Zhang, Jiaqi Sun, Lei Chen, Kangrong Lu, Liping Cao, Xuemei Liu

**Affiliations:** ^1^CAS Key Laboratory of Brain Connectome and Manipulation, The Brain Cognition and Brain Disease Institute, Shenzhen Institute of Advanced Technology, Chinese Academy of Sciences, Shenzhen-Hong Kong Institute of Brain Science-Shenzhen Fundamental Research Institutions, Shenzhen, Guangdong, China; ^2^The Affiliated Brain Hospital of Guangzhou Medical University, Guangzhou, Guangdong, China; ^3^Key Laboratory of Neurogenetics and Channelopathies of Guangdong Province and the Ministry of Education of China, Guangzhou Medical University, Guangzhou, Guangdong, China; ^4^Southern Medical University, Guangzhou, Guangdong, China; ^5^School of Basic Medical Sciences, Southern Medical University, Guangzhou, Guangdong, China; ^6^University of Chinese Academy of Sciences, Beijing, China

**Keywords:** bipolar disorder, major depressive disorder, adolescent, diagnosis, bibliometric, clinical features, biological biomarkers

## Abstract

**Background:**

Mood disorders are very common among adolescents and include mainly bipolar disorder (BD) and major depressive disorder (MDD), with overlapping depressive symptoms that pose a significant challenge to realizing a rapid and accurate differential diagnosis in clinical practice. Misdiagnosis of BD as MDD can lead to inappropriate treatment and detrimental outcomes, including a poorer ultimate clinical and functional prognosis and even an increased risk of suicide. Therefore, it is of great significance for clinical management to identify clinical symptoms or features and biological markers that can accurately distinguish BD from MDD. With the aid of bibliometric analysis, we explore, visualize, and conclude the important directions of differential diagnostic studies of BD and MDD in adolescents.

**Materials and methods:**

A literature search was performed for studies on differential diagnostic studies of BD and MDD among adolescents in the Web of Science Core Collection database. All studies considered for this article were published between 2004 and 2023. Bibliometric analysis and visualization were performed using the VOSviewer and CiteSpace software.

**Results:**

In total, 148 publications were retrieved. The number of publications on differential diagnostic studies of BD and MDD among adolescents has been generally increasing since 2012, with the United States being an emerging hub with a growing influence in the field. Boris Birmaher is the top author in terms of the number of publications, and the *Journal of Affective Disorders* is the most published journal in the field. Co-occurrence analysis of keywords showed that clinical characteristics, genetic factors, and neuroimaging are current research hotspots. Ultimately, we comprehensively sorted out the current state of research in this area and proposed possible research directions in future.

**Conclusion:**

This is the first-ever study of bibliometric and visual analyses of differential diagnostic studies of BD and MDD in adolescents to reveal the current research status and important directions in the field. Our research and analysis results might provide some practical sources for academic scholars and clinical practice.

## 1. Introduction

Mood disorders are highly prevalent among adolescents, with an estimated lifetime prevalence ranging from 10.7 to 17.3% (Merikangas et al., 2010; Copeland et al., 2011; Ormel et al., 2015; Solmi et al., 2021). The main risk factors for adolescent emotional disorders include both biological and environmental factors. Biological factors mainly refer to genetic predisposition, while environmental factors mainly include psychological and social factors, such as stress and trauma (DelBello, 2018; Kalin, 2021). Mood disorders interfere with all aspects of the lives of affected adolescents, including impaired cognitive functioning, increased risk of psychiatric hospitalization, substance use disorders, family dysfunction, deficits in educational performance, and more (Carlson and Kashani, 1988; Lewinsohn et al., 1998). Mood disorders are also a major risk factor for adolescent suicide, which remains the second-most common cause of death among youth (Kessler, 2012; Nock et al., 2013; Bauer et al., 2018). Considering its enormous burden on patients and society, early standardized treatment of patients with mood disorders is extremely necessary.

**Figure 1:**
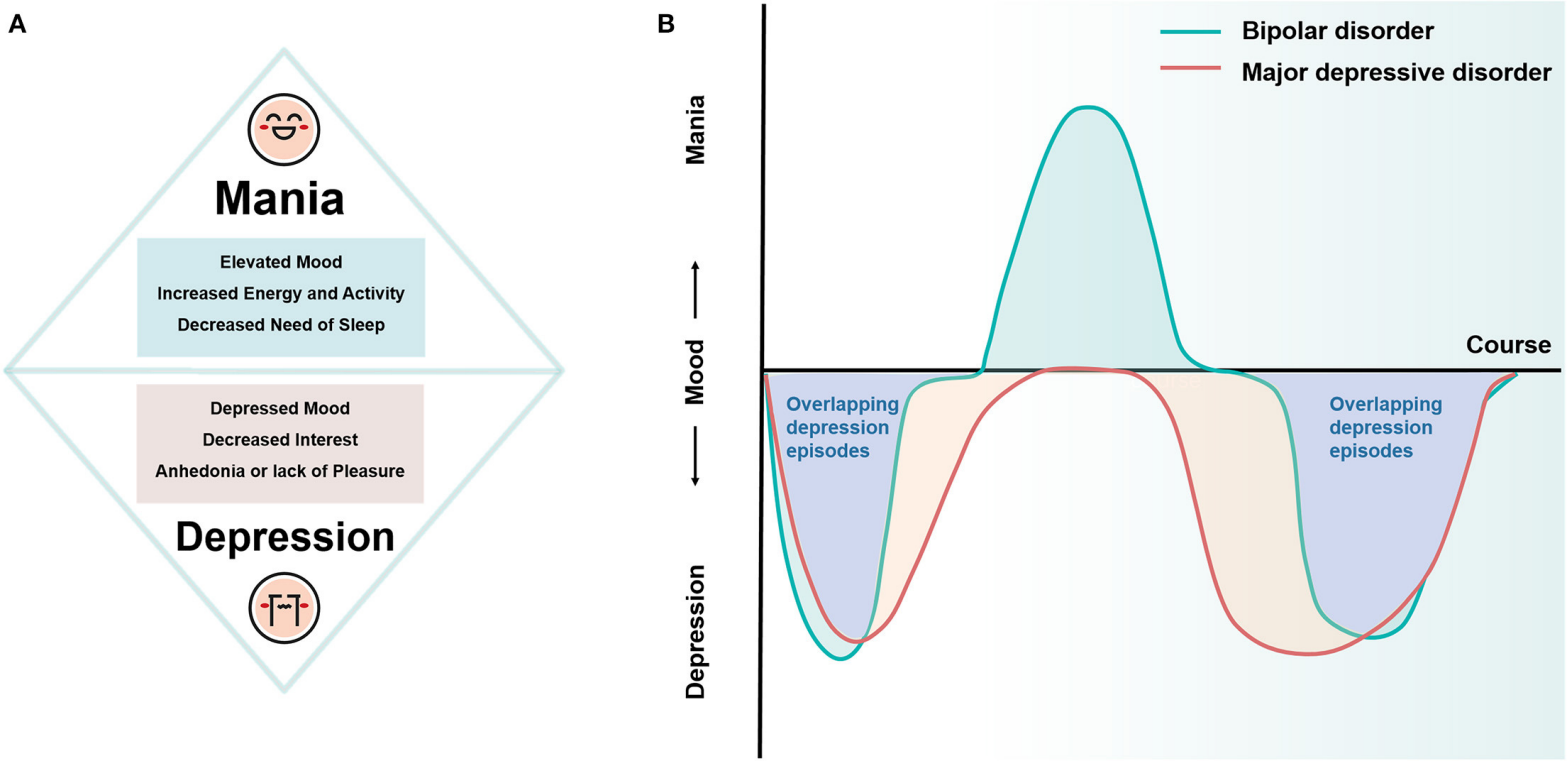
Diagrammatic drawing of the symptoms and course of BD and MDD. **(A)** Core symptoms of mania and depression episodes. **(B)** Patients with BD often present depression episodes as the first symptom, and they dominate the subsequent course of the disease. The overlapping depression episodes in BD and MDD pose a significant challenge in the clinical differential diagnosis. BD, bipolar disorder; MDD, major depressive disorder.

Bipolar disorder (BD) and major depressive disorder (MDD) are two of the most common mood disorders. BD is defined by mood instability and is diagnosed based on the recurrent occurrence of alternating manic or hypomanic and depressive episodes (Grande et al., 2016). Manic/hypomanic episodes are typified by elevated mood, increased energy and activity, and decreased need to sleep, and depression episodes are characterized by depressed mood, decreased interest, and anhedonia or lack of pleasure (Otte et al., 2016), while MDD presents as depression episodes only and has the same diagnostic criteria as bipolar depression episodes (hereafter referred to as unipolar and bipolar depression, respectively) (Figure 1A). Although depression episodes and mania/hypomania episodes are easy to distinguish, previous studies suggest that the majority of BD patients present with depression episodes rather than manic episodes as their first symptom (Lish et al., 1994; Perlis et al., 2004) and that depression episodes predominate in the subsequent course of the disease (Altshuler et al., 2010). The overlapping depressive symptoms in BD and MDD pose a great challenge to the realization of rapid and accurate clinical diagnosis (Belmaker and Agam, 2008; Hirschfeld, 2014) (Figure 1B). It is estimated that about 50–75% of BD patients were initially misdiagnosed as MDD (Hirschfeld et al., 2003; Tondo et al., 2014), with an average misdiagnosis time of 10 years, even longer for young BD (Godwin and Jam, 2008), resulting in about one-third of BD patients not receiving appropriate treatment (Ghaemi et al., 1999). Antidepressants are the first-line treatment for depressive episodes, but in patients with bipolar depression, antidepressants carry a significant risk of inducing manic episodes or hypomanic episodes and may also increase the risk of suicidal behavior and cause adverse consequences of chronicity in the course of BD, especially among adolescents (Faedda et al., 2004; Akiskal and Benazzi, 2005; Baldessarini et al., 2005; Perlis et al., 2011), all of which may lead to a poorer ultimate clinical and functional prognosis and further burden the individual, family, and society. Therefore, differentiating between BD and MDD in adolescents is a challenging but necessary research topic. Successful discrimination of these two disorders will not only help in diagnostic decisions to ensure optimal clinical and functional outcomes for all individuals but also provide insight into their etiology and neuropathological processes.

Since the specific pathogenesis of BD and MDD is still unclear, no relevant articles have been published to sort this field, leading to a lack of clear understanding and good clinical application. Bibliometrics is an emerging and comprehensive analytical approach that can fill this gap by analyzing publications qualitatively to assess the contributions of countries, institutions, and authors in the field and to explore and suggest important research directions and hotspots in depth (Wang et al., 2021). This study aims at exploring the current research status and important directions in the field of differential diagnostic studies of BD and MDD in adolescents through bibliometric and visual analysis to provide some practical sources for academic scholars and clinical practice.

## 2. Materials and methods

### 2.1. Data collection

Web of Science (WoS) is one of the world's most comprehensive and authoritative databases, providing access to over 12,000 important high-quality journals from around the world and their bibliometric indicators, and is widely used for bibliometric analysis and visualization of the scientific literature (Wu et al., 2021). So the WoS Core Collection database was chosen as the source for data retrieval.

**Figure 2:**
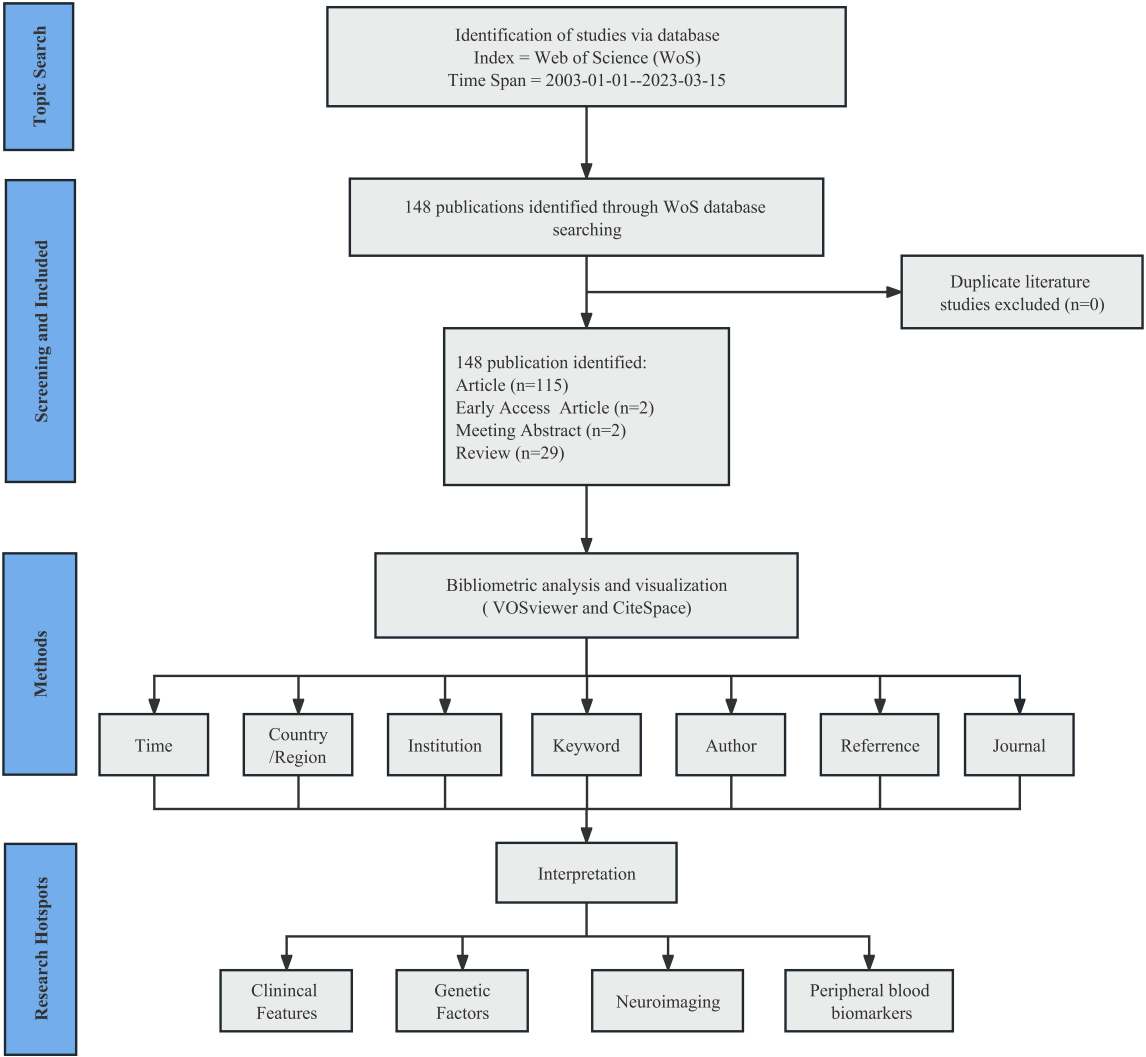
Flowchart of literature screening included in this study.

For data retrieval, we applied the advanced retrieval function of the database with the following formulae: [ALL = (bipolar disorder) OR ALL = (BD) OR ALL = (bipolar depression)] AND [ALL = (MDD) OR ALL = (unipolar depression) OR ALL = (major depressive disorder)] AND [ALL = (discriminate) OR ALL = (discrimination) OR ALL = (differentiate) OR ALL = (identification)] AND [ALL = (adolescent) OR ALL = (youth) OR ALL = (teenager)]. The index was restricted to SCI-EXPANDED. Language is not a limitation. In total, 115 articles, two early access articles, two meeting abstracts, and 29 reviews fulfilled the criteria. Finally, we selected the export option “Full Record and Cited References” and exported all retrieved documents in plain text format (Figure 2).

### 2.2. Duplication and visualization

CiteSpace version 6.2.R2 was chosen to analyze our data. Prior to analysis, the literature was de-duplicated using the WOS de-duplication tool. The setting parameters were as follows: The time slice was set to 2004–2023, and the one-time slice was set to each year. Title, abstract, and keywords were selected as the term sources. The pruning options were Pathfinder and Pruning sliced networks. Other parameters were set to default values. After this process, 148 valid studies remained. Based on the different types of nodes to be studied, visual graphs had to be generated. Authors, institutions, and countries were selected for collaborative network analysis. Keywords were selected for the coexistence analysis. A co-cited reference analysis was also carried out. Visualization was performed by VOSviewer (version 1.6.16) and CiteSpace 6.2R2.

## 3. Results

### 3.1. Distribution of publication dates

**Figure 3:**
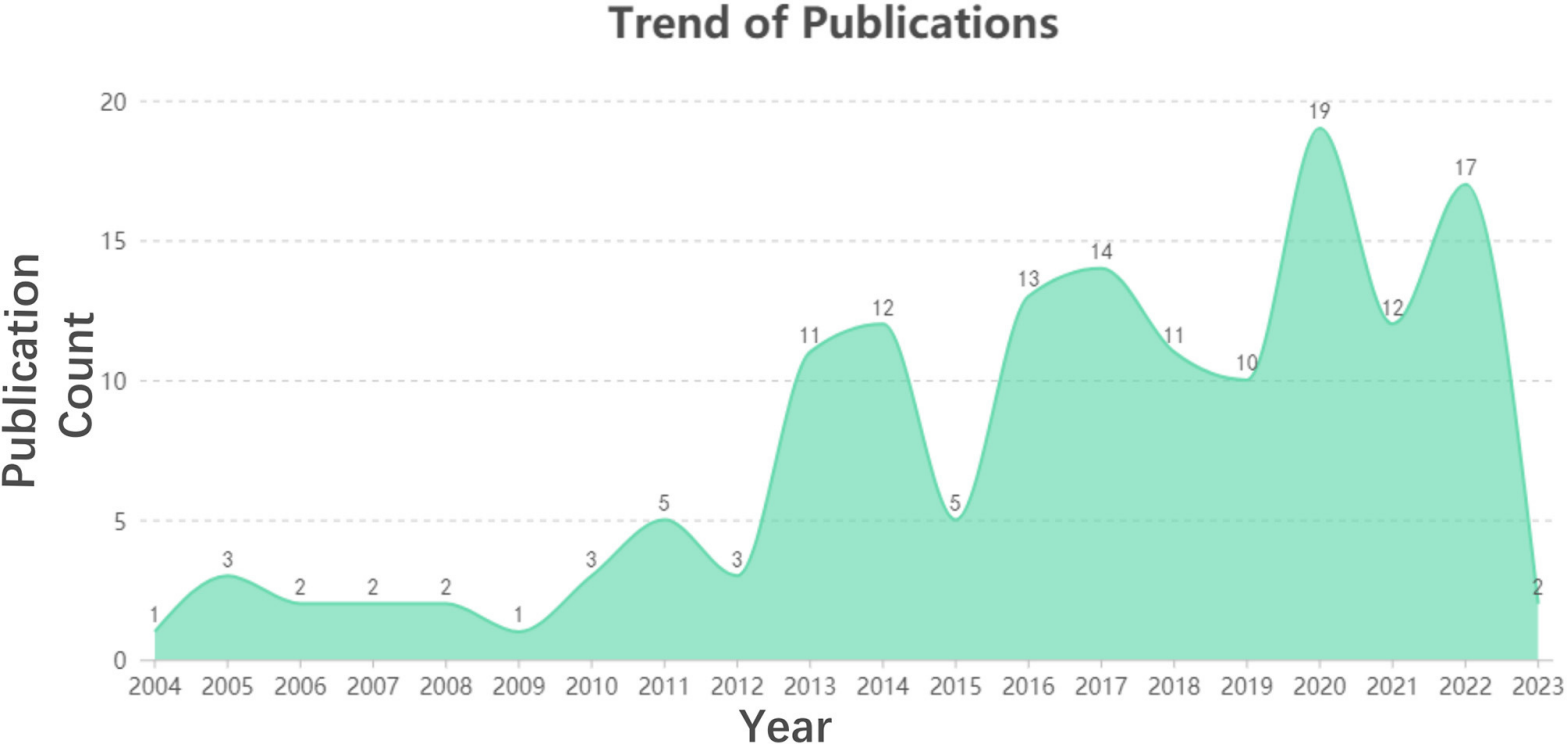
Time distribution of publications on differentiation between BD and MDD in adolescents.

The number of articles published each year represents the development of a research area. The number of publications on the distinction between BD and MDD in adolescents fluctuates between 2004 and 2023, with an overall increasing trend. A total of 48 studies were published between 2020 and 2022, accounting for 32.43% of the total literature for the selected time period (Figure 3).

### 3.2. Network analysis of the national distribution and collaboration

**Figure 4:**
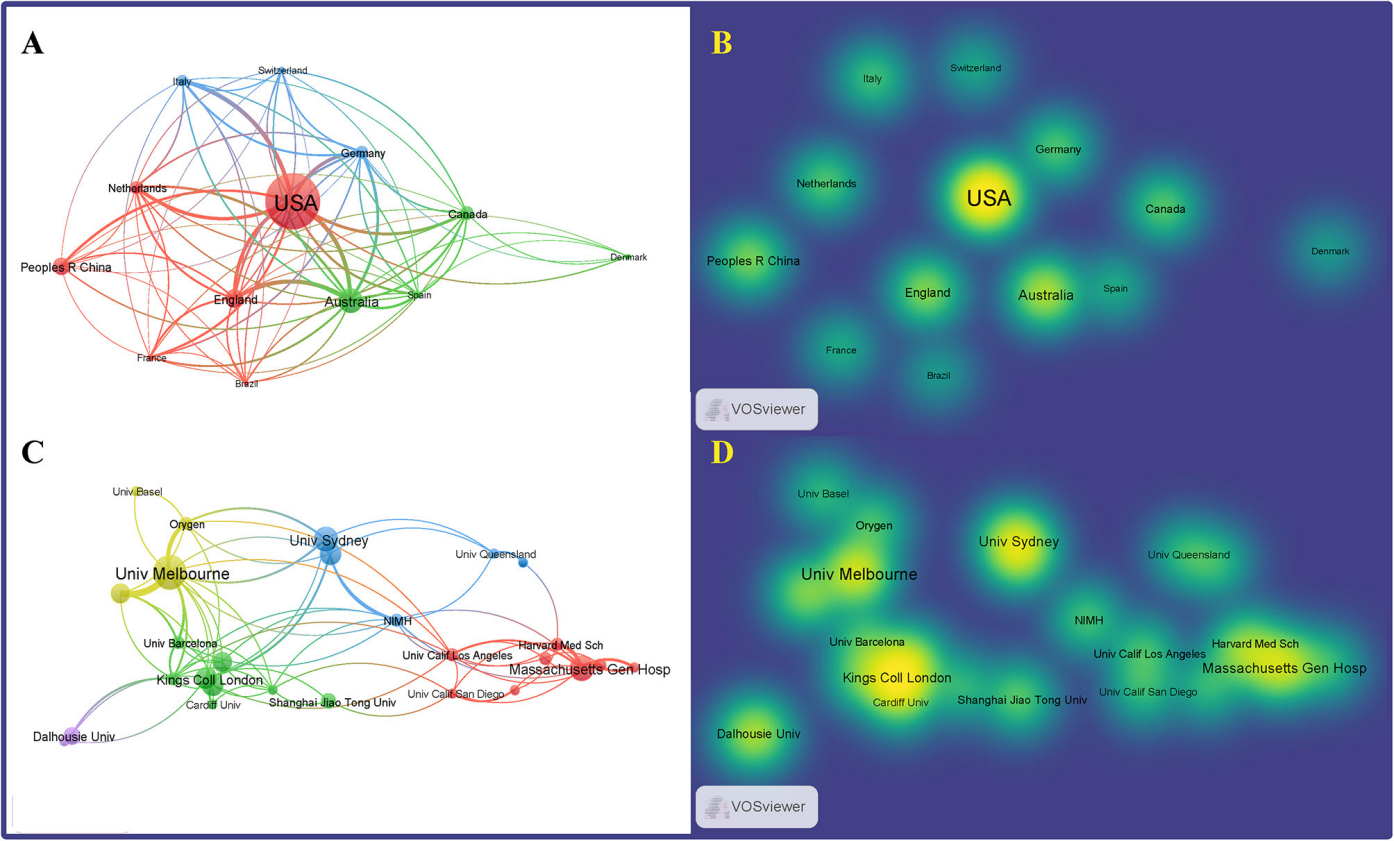
Distribution of publications and national research collaborations regarding differentiation between BD and MDD in adolescents. Network **(A)** and density **(B)** mapping the contributions of each country or region to publications. Network **(C)** and density **(D)** map, generated by VOSviewer, depict collaborations among institutions.

VOSviewer was used to map the international collaboration network. In the density visualization (Figures 4A, B), the higher the number of publications of a node (representing a country), the larger the font of the label, while the closer the collaboration between two nodes, the more similar their color. The United States had the largest circle and the largest font, indicating that it has made the most significant contribution to research on the differentiation between BD and MDD in adolescents. Countries, such as Italy, England, and China also made significant contributions, as indicated by the size of the node. The CiteSpace software concisely calculated and visualized the centrality and publications of each country (Table 1). The United States, England, Italy, and China were the top four countries that contributed to publications in this area. Among them, the United States, the largest node on the map, published 73 studies, which was significantly higher than the others, indicating that the research strength was far ahead. Centrality was an index to measure the importance of a node in the network in CiteSpace. Thus, the closer the connection to the other nodes, the larger the purple circle (Chen, 2006; Li and Chen, 2016). Based on the analysis of the number and centrality of published articles, China ranked fourth with 22 publications. However, the centrality was zero, suggesting that China needs to strengthen international communication and cooperation to improve its international influence in this field. The top 10 countries in terms of publication volume and their centrality are shown in Table 1.

**Table 1:** List of the top countries and research institutions by the number of publications and centrality.

**Country ranking**				**Institution ranking**			
**Rank**	**Country**	**Publication count**	**Centrality**	**Rank**	**Institution**	**Publication count**	**Centrality**
1	USA	73	0.53	1	University of Melbourne	15	0.07
2	Australia	32	0.08	2	King's College London	12	0.28
3	England	24	0.09	3	Deakin University	10	0.08
4	People's Republic of China	22	0.00	4	The University of Sydney	9	0.02
5	Canada	17	0.22	5	Massachusetts General Hospital	8	0.11
6	Germany	16	0.08	6	Newcastle University	8	0.05
7	Netherlands	16	0.06	7	University of Pittsburgh	8	0.1
8	Italy	14	0.16	8	Stanford University	7	0.05
9	France	9	0.11	9	Harvard Medical School	7	0.22
10	Spain	9	0.18	10	Dalhousie University	7	0.06

### 3.3. Research institutional cooperation network analysis

Visualization maps can directly display the distribution of research forces in the field. Moreover, the close cooperation between institutions facilitates the development of the discipline and the sharing of outcomes. The software VOSviewer was used to generate a map of the cooperative network of research institutions (Figures 4C, D). The top four research institutions with publications on differentiation between BD and MDD in adolescents were the University of Melbourne, King's College London, Deakin University, and the University of Sydney. The 10 research institutions in terms of publication volume are presented in Table 1.

### 3.4. Analysis of the author collaboration network

Bibliometrics can provide information on outstanding researchers and related research groups in this field to strengthen communication and cooperation within the discipline. The author's collaboration was analyzed using the software CiteSpace 6.2R2. The top three authors in terms of publication volume were Boris Birmaher, Jan Scott, and Michael Berk (Table 2). These authors are active and have had a strong influence on their respective disciplines. The author with the most published articles and the highest centrality was Boris Birmaher (University of Pittsburgh, USA).

**Table 2:** List of the top seven authors based on publication volume and centrality.

**Rank**	**Author**	**Publication count**	**Centrality**
1	Birmaher, Boris	7	0.05
2	Scott, Jan	7	0.02
3	Berk, Michael	7	0.02
4	Correll, Christoph U	5	0.03
5	Hickie, Ian B	4	0.02
6	Biederman, Joseph	4	0
7	Axelson, David	4	0

### 3.5. Reference co-occurrence analysis

The software CiteSpace 6.2 R2 was used to analyze, and VOSviewer was used to visualize. Among the references, the top five articles cited by the 148 publications are listed in Table 3. We also apply the cluster view in CiteSpace for reference occurrences. The most cited reference is “The predictive validity of bipolar at-risk (prodromal) criteria in help-seeking adolescents and young adults: a prospective study,” written by Bechdolf et al. (2014) and published in *Bipolar Disorders*. The study “Diagnostic precursors to bipolar disorder in offspring of parents with bipolar disorder: a longitudinal study,” established by Axelson et al. (2015) was published in the *American Journal of Psychiatry* in 2015.

**Table 3:** List of the highly cited references.

**Ranking**	**Publication**	**Citation**	**Centrality**	**Year**
1	The predictive validity of bipolar at-risk (prodromal) criteria in help-seeking adolescents and young adults: a prospective study	7	0.11	2014
2	Diagnostic precursors to bipolar disorder in offspring of parents with bipolar disorder: a longitudinal study	6	0.17	2015
3	Course of subthreshold bipolar disorder in youth diagnostic progression from bipolar disorder not otherwise specified	6	0.02	2011
4	A 16-year prospective study of prodromal features prior to BPI onset in well Amish children	6	0.03	2012
5	Risk constellations prior to the development of bipolar disorders: rationale of a new risk assessment tool	6	0.01	2012

### 3.6. Journal and cited journal analysis

All 148 publications on differentiation between BD and MDD in adolescents have been cited in 340 journals. The top journals and co-cited journals are listed in Table 4. The most published journal is the *Journal of Affective Disorders* (25), and the most cited journal is the *American Journal of Psychiatry* (127). Most journals and co-cited journals have high impact factor (IF) and are in Q1 based on the Journal Citation Reports (JCR).

**Table 4:** List of the top 14 journals and co-cited journals.

**Ranking**	**Journal**	**Output**	**IF**	**JCR**	**Co-cited journal**	**Citation**	**Centrality**	**IF**	**JCR**
1	*Journal of Affective Disorders*	25	6.533	Q1	*American Journal of Psychiatry*	127	0.13	19.242	Q1
2	*Frontiers In Psychiatry*	8	5.435	Q2	*Journal of Affective Disorders*	115	0.02	6.533	Q1
3	*Bipolar Disorders*	6	5.345	Q1/Q2	*Archives of General Psychiatry*	112	0.07	-	-
4	*Psychiatry Research Neuroimaging*	6	2.493	Q3/Q4	*Biological Psychiatry*	96	0.15	12.81	Q1
5	*Biological Psychiatry*	5	12.81	Q1	*Bipolar Disorders*	92	0.02	5.345	Q1/Q2
6	*Psychological Medicine*	5	10.592	Q1	*Psychological Medicine*	92	0.02	10.592	Q1
7	*Schizophrenia Bulletin*	4	7.348	Q1	*British Journal of Psychiatry*	86	0.03	10.671	Q1
8	*Translational Psychiatry*	4	7.989	Q1	*Journal of Clinical Psychiatry*	84	0.04	5.906	Q1/Q2
9	*BMC Psychiatry*	3	4.144	Q2	*Journal of the American Academy of Child and Adolescent Psychiatry*	84	0.01	13.113	Q1
10	*Early Intervention in Psychiatry*	3	2.72	Q3	*Molecular Psychiatry*	73	0.03	13.473	Q1
11	*European Child Adolescent Psychiatry*	3	5.349	Q1/Q2	*Acta Psychiatrica Scandinavica*	66	0.13	7.734	Q1
12	*JAMA Psychiatry*	3	25.911	Q1	*Psychiatry Research*	62	0.03	11.225	Q1
13	*Journal of Clinical Psychiatry*	3	5.906	Q1/Q2	*Lancet*	56	0.03	202.73	Q1
14	*Journal of Psychiatric Research*	3	5.25	Q2	*Journal of Psychiatric Research*	55	0.02	5.25	Q2

IF, impact factor; JCR, sections divided in terms of journal citation reports.

### 3.7. Keyword co-occurrence analysis

The frequency of keywords can reflect the hotspots in a field of research. Moreover, the occurrence of keywords in different periods can reflect the development and changes in a research field. The software CiteSpace 6.2R2 was used to analyze the co-occurrence of keywords. Apart from the search terms, the top five keywords based on occurrence frequency were “follow up”, “risk factor” and “meta-analysis”. And the top four keywords according to centrality were “comorbidity”, “spectrum disorder”, “risk factor” and “genome wide association” (Table 5).

**Table 5:** Top keywords based on occurrence frequency and centrality.

**Ranking**	**Keyword**	**Occurrence**	**Ranking**	**Keyword**	**Centrality**
1	Bipolar disorder	94	1	Bipolar disorder	0.46
2	Major depressive disorder	57	2	Children	0.28
3	Adolescent	25	3	Adolescent	0.24
4	Children	24	4	Major depressive disorder	0.18
5	Schizophrenia	18	5	Co-morbidity	0.17
6	Major depression	15	6	Major depression	0.16
7	Mood disorder	14	7	Schizophrenia	0.13
8	Follow-up	14	8	Childhood	0.1
9	Psychiatric disorder	10	9	Spectrum disorder	0.09
10	Unipolar depression	10	10	Mood disorder	0.08
11	Risk factor	10	11	Risk factor	0.08
12	Meta-analysis	10	12	Genome-wide association	0.08

**Figure 5:**
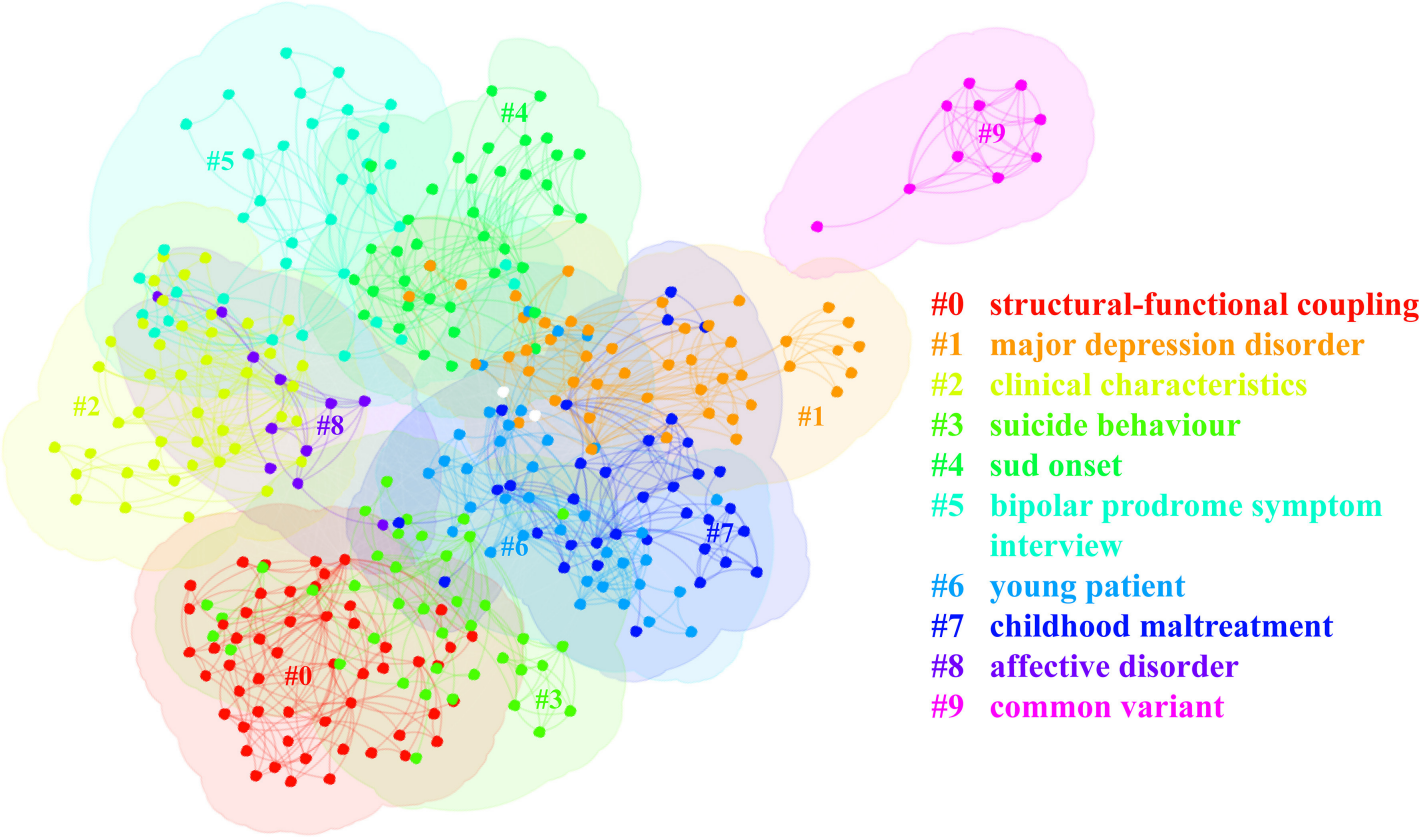
Co-occurrence analysis of keywords about distinguishing bipolar and unipolar depression. SUD, substance use disorder.

Then we clustered these co-occurring keywords. The keywords of research formed 10 clusters, representing 10 main research directions (Table 6). The silhouette value (an indicator reflecting the internal homogeneity of the cluster; the closer it is to 1, the higher the homogeneity) and size of each cluster are in reasonable context, indicating the cluster division is qualified (Chen et al., 2014). The visualized maps generated by the analysis of the visualization of the clustered keywords can provide the researcher with an indication of what to study regarding the distinction between adolescent BD and MDD. Labels for clusters were extracted by keyword, title, and abstract and the retrieved words were replaced by meaningful labels (Figure 5). Apart from the search words such as #1 (major depression disorder), #6 (young patient), and #8 (affective variant), cluster #0 (structural-functional coupling), #2 (clinical characteristics), #3 (suicide behavior), #5 (bipolar prodrome symptom interview), #7 (childhood maltreatment) and #9 (common variant) suggest important directions in the identification of BD and MDD in adolescents. By interpreting the keywords about each cluster, clusters #2, #3, #5, and #7 can be classified as having clinical characteristics. In addition, #9 suggests that genetic factors, especially common variants, are also an important direction, and #0 suggests that neuroimaging research is another hot topic. Due to the complexity of the keyword and the undefined label, the significance of cluster #4 is not clear.

**Table 6:** Information about keyword clusters.

**Cluster ID**	**Size**	**Silhouette**	**Mean year**	**Label by keyword**	**Label by title**	**Label by abstract**	**Top terms (LLR)**
#0	54	0.763	2016	Connectivity	Bipolar disorder patient	Structural functional coupling	Mixed features; C-reactive protein; immune dysregulation; symptom; atypical symptoms
#1	47	0.874	2011	Major depressive disorder	Bipolar disorder	Bipolar patient	Bipolar I disorder; unified protocol; health; gender; course
#2	44	0.862	2013	Mood disorder	Clinical characteristics	Young patient	Onset; mood disorder; parent; precursor; suicidal ideation
#3	40	0.755	2012	Suicidal behavior	Adolescent suicide attempt	Single diagnostic categories	Depression; epidemiology; bipolar ii disorder; subsyndromal bipolar disorder; criteria
#4	39	0.739	2011	Substance use disorder	OCD-affected sibling pair	SUD onset	Adolescent; amygdala volume; co-morbidity survey replication; adolescence; case finding
#5	37	0.822	2015	Clinical high risk	Bipolar prodrome symptom interview	Cronbach's alpha	Adolescent; major depressive disorder; treatment enhancement program; risperidone; early-onset bipolar disorder
#6	36	0.882	2010	Cyclothymic temperament	Young patient	Young patient	Anxiety; affective lability; prodrome; cognitive functioning; mental imagery
#7	35	0.78	2014	Autism spectrum disorder	Pattern recognition	Childhood maltreatment	DSM-5; mixed states; prediction; depressive onset; major depression
#8	11	0.911	2015	Neurocognitive tests	Affective disorder	Cognitive function	Prevention; mortality; pediatric; parenting; somatic morbidity
#9	10	0.971	2012	Na+/H+ exchanger	Common variant	Intracranial volume	Catatonia; suicidal; electroconvulsive therapy; mixed; severity

## 4. Discussion

Bibliometrics, a research branch of library and information science, mainly uses mathematical methods to quantitatively analyze relationships between documents (Ye, 2005). This study used CiteSpace and VOSviewer to describe and visually present annual publications, countries, institutions, influential authors, and journals in the differentiation between BD and MDD in adolescents from the earliest literature to March 2023, to understand the research hotspots, frontiers, and trends in the field. Our research and analysis results might provide some practical sources for academic scholars and clinical practice.

### 4.1. General state of study on differential diagnosis between BD and MDD in adolescent

In total, 148 publications were retrieved. Our analysis showed that the number of publications on differential diagnostic studies of BD and MDD among adolescents has been generally increasing since 2012, with the United States being an emerging hub with a growing influence in the field. We speculate that this may be linked to the country's level of development and economic prosperity, suggesting that the economic status of a country plays a crucial role in shaping research progress. It is worth noting that China, as the fourth most prolific country in terms of publications, has conducted a significantly greater amount of research in this field in recent years. However, its centrality score is zero, indicating that China should enhance international communication and cooperation to boost its global influence in this area. Most of the top institutions were colleges and universities, suggesting that these institutions are the main platforms for research groups. Boris Birmaher is the top author in terms of number of publications and contributions to the field.

The *Journal of Affective Disorders* is the most prolific publication in this field, covering a wide range of topics related to affective disorders, including research on neuroimaging, cognitive neurosciences, genetics, molecular biology, and experimental and clinical neurosciences. Its comprehensive coverage and significant contributions make it a leading resource for all aspects of affective disorders.

In this study, the most frequently cited reference was written by Bechdolf et al. (2014). In this study, they developed a set of criteria called “ultra-high-risk criteria for bipolar disorder” [bipolar at-risk (BAR)], which supports the potential for identifying individuals before the onset of mania/hypomania.

A longitudinal study conducted by Axelson et al. (2015), titled “Diagnostic Precursors to Bipolar Disorder in Offspring of Parents with Bipolar Disorder,” was published in the *American Journal of Psychiatry* in 2015 and is currently one of the second-most cited articles in its field. The study aimed to identify diagnostic risk factors for manic, mixed, or hypomanic episodes in the offspring of parents with BD. Findings revealed that subthreshold manic or hypomanic episodes were diagnostic risk factors for developing these episodes, thereby indicating a significantly higher risk of BD in those individuals. The aforementioned study supports the possibility of early identification of BD patients, which has significant implications for both research directions and clinical practice.

### 4.2. Current research hotspots and frontiers in future

Co-occurrence analysis of keywords showed that clinical characteristics, genetic factors, and neuroimaging are current research hotspots in this field. Subsequently, we examine the hotspots that emerged from our analysis one by one.

#### 4.2.1. Clinical features

At present, psychopathology is still the primary evidence for the diagnosis of mental disorders. Although both BD and MDD experience depression episodes, their different clinical concomitant features may be indicative of a clinical diagnosis. Therefore, the purpose of this part of the literature was to review which clinical features indicate bipolar depression rather than unipolar depression in adolescents.

First, several studies have consistently found that BD is associated with more psychiatric co-morbidities than MDD, including conduct disorder, obsessive-compulsive disorder, substance abuse, and borderline personality disorder (Wozniak et al., 2004; Diler et al., 2017; Cui et al., 2022).

Second, atypical features, including increased appetite or sleep, psychomotor retardation, and mood reactivity, have long been considered reliable markers for identifying BD. In a study conducted by Cui et al. (2022) on Chinese inpatients with depressive episodes, a significantly higher proportion of adolescents with bipolar depression were found to have atypical features than patients with unipolar depression, but this study did not specify the definition of atypical symptoms. A case–control study by Diler et al. (2017) found that the reactivity of mood [odds ratio (OR) = 9.7] and daytime sleepiness (OR = 4.1) was significantly different and independently correlated between groups with BD and MDD, while there was no significant difference in increased appetite and weight loss between groups. This suggests that although atypical features are a potential risk factor for BD, not all atypical symptoms are helpful in the identification of BD.

Subsyndromal manic symptoms are more common in bipolar depression and are characterized by elation, exaggeration, verbal stress, quick thinking, wandering thoughts, inattention, hyperactivity, abnormal energy symptoms, inappropriate laughter, and acute/creative thinking. Compared with youth with unipolar depression, patients with BD had significantly more severe manic symptoms (Diler et al., 2017). In addition, patients with bipolar depression had significantly higher scores on the Hypomania Checklist-32 Questionnaire than patients with unipolar depression (Nisha et al., 2015; Cui et al., 2022). This suggests that although patients complain of depression, clinicians still need to carefully screen for manic-related symptoms to identify the possibility of BD at an early stage.

Several studies among adult samples have consistently found that patients with BD have more psychotic symptoms during episodes of depression than patients with MDD (Gan et al., 2011; Mitchell et al., 2011; Dervic et al., 2015), but in the adolescent sample, the results are inconsistent. Diler et al. (2017) found depressed BD youth showed higher rates of psychotic symptoms (40% vs. 8.6%) in adolescents by comparing the unipolar depression group with the bipolar depression group. However, this result could not be repeated in another study that included 32 patients with depression and 53 with BD (Cui et al., 2022), which may be due to the depression sample included in the first study being too young and psychiatric symptoms not being well screened, which needs to be further verified in a larger sample.

A high suicide rate is a prominent feature of BD (McIntyre et al., 2020). Approximately 15–20% of patients with BD eventually die by suicide, and a history of suicide attempts is considered to be a predictor of BD (Inoue et al., 2015). In addition to suicide, non-suicidal self-injury is also one of the serious symptoms of patients with mood disorders in adolescents. A number of studies have consistently pointed out that higher rates of suicidality and non-suicidal physical self-injurious acts were found in bipolar depression subjects compared to unipolar depression subjects (Wozniak et al., 2004; Diler et al., 2017; Cui et al., 2022). The reason for this difference may be that the psychosocial function of adolescents with BD depression is more impaired than that of patients with MDD, including school performance, peer relationships, family relationships, and many other areas (Wozniak et al., 2004).

In addition, a family history of psychiatric diseases is one of the more distinctive differential features of BD and MDD. Clinical studies have confirmed that the positive family history like mania/anxiety disorders in MDD is much lower than that in BD (Wozniak et al., 2004; Scott et al., 2013; Shon et al., 2014). Therefore, family history is an important factor, but it cannot be simply inferred because the prevalence of MDD is much higher than that of BD.

Overall, BD is more likely to exhibit more psychiatric co-morbidity, atypical features, psychotic features, risk of self-inflicted suicide, subsyndromal manic symptoms, and a high rate of positive family history. However, the extent to which we can rely on these clinical features to distinguish BD from MDD remains to be seen because the incidence of these clinical features generally varies little, with low specificity for diagnostic purposes of identification (Akiskal and Benazzi, 2008). For diagnostic purposes of identification, a symptom-oriented diagnosis strategy alone is not enough to help recognize bipolar depression and unipolar depression. In addition, these clinical features may be inaccurate, subject to some recall bias, and can only be defined by assessment after the presentation, thus limiting their use in the early stages of the disease (Van Meter et al., 2016). Therefore, objective diagnostic biomarkers are needed.

#### 4.2.2. Genetic factors

Genetic background plays a crucial role in mental disorders. Studies suggest that depressive disorder has a heritability of only 30–50% (Sullivan et al., 2000; Kendler et al., 2006, 2018; Polderman et al., 2015), while the heritability of BD is up to 60–80% (Johansson et al., 2019; Fabbri, 2021). Early pedigree studies suggested that MDD demonstrated familial aggregation, with first-degree relatives having a 1.84-fold higher risk of developing depressive disorders compared to the general population (Sullivan et al., 2000). A meta-analysis of adolescents with bipolar I disorder showed that their first-degree relatives had a 5.96-fold higher risk of bipolar I disorder than the average population (Wozniak et al., 2012). These findings indicate that BD, especially in adolescents, has a higher heritability, greater genetic vulnerability, and stronger familial aggregation compared to depressive disorders (Faraone et al., 2003; Wozniak et al., 2012). Thus, theoretically, we could use genetic biomarkers to provide complementary diagnostic information in the early stages, when clinical information is uncertain.

In general, common variants are polymorphic variations that are expected to occur at a frequency >1% and have a minor allele frequency >5%. In contrast, the frequency of rare variants ranged from 0.1 to 1% (Bodmer and Bonilla, 2008). Combining the #9 common variant shown by co-occurrence analysis of keywords (Figure 5) with the low frequency of rare variants and their small contribution to the overall genetic susceptibility to the disease, the discussion here will mainly focus on common variants. With the rapid development of genome-wide association studies (GWAS), more than 60 genetic risk loci have been identified in BD (Mullins et al., 2021) and more than 100 genetic risk loci in MDD to date (Howard et al., 2019). However, based on the polygenic genetic basis of BD and MDD, the number of single nucleotide polymorphism (SNP) constituting disease risk is very large (Purcell et al., 2009), and the effect value of each susceptible SNP is very small (Gandal et al., 2016), which makes it difficult to apply individual SNP mutations as diagnostic aids in the clinical setting. Using the results of the large-sample GWAS studies, we can calculate the individual polygenic risk score (PRS) (Choi et al., 2020) associated with a specific disease by summarizing the situation of a large number of individual SNP mutations. A study investigating the development of BD in individuals with an early diagnosis of unipolar depression (age 10–35 years at first diagnosis of MDD) showed that only the PRS for BD predicted the transition from unipolar depression to BD (Musliner et al., 2020), suggesting that the BD PRS may differentiate BD from MDD in the early stage of the disease. During the same period, Liebers et al. (2021) also found that BD PRS may serve to discriminate between BD and MDD among adult samples to some extent in patients with extreme distribution, whereas MDD PRS cannot.

These findings imply that genetic differences between BD and MDD, by identifying a significant number of genetic risk variant loci for each disorder, could serve as a reliable and powerful method to distinguish between them in future. However, there is a lack of literature on the early identification of BD and MDD in adolescents using PRS. Addressing this gap should be a priority for future research in this field.

#### 4.2.3. Neuroimaging

Much progress has been made in recent years in the development of neuroimaging. Magnetic resonance imaging (MRI) can offer detailed information on brain structure and function or even metabolites via structural MRI, functional MRI (fMRI), magnetic resonance spectroscopy (MRS), or other technologies. These different imaging data are used to calculate different imaging indicators, which can be used as biological markers for disease classification and prognosis prediction. Therefore, further investigation of the neuroimaging characteristics of adolescents with BD or MDD will be important.

Computing whole-brain functional connectivity (FC) by resting-state fMRI data, Goldman et al. (2022) found that it could be used as a marker to distinguish unipolar depression from bipolar depression in adolescents. They observed that bipolar depression showed increased interhemispheric FC between frontal areas but not in unipolar depression.

A study conducted by MacMaster et al. (2008) investigated the pituitary gland size of individuals aged 13–20 with major depressive disorder (MDD) and bipolar disorder (BD) during a depressive episode. The study aimed to investigate abnormal development in the hypothalamic–pituitary–adrenal (HPA) axis in affective disorders. The authors found that both unipolar and bipolar depression patients had larger-than-normal pituitary glands, which they suggested may be caused by endocrine disorders in individuals with mood disorders. However, no significant differences were found between those with unipolar and bipolar depression. With this team, imaging studies were again performed in 2014 on a group of 11–19 years old with affective disorders. In this study, they found that the right side of the anterior cingulate cortex (ACC) white matter was smaller in unipolar depression than in bipolar depression, and the right side of the ACC gray matter also showed a tendency to decrease (MacMaster et al., 2014).

Comparably, Shi et al. (2014) used MRS to find that metabolic levels of ACC differed in adolescents with bipolar depression versus unipolar depression. Compared to bipolar depression, unipolar depression showed a significantly increased ACC choline/creatine ratio, which may reflect non-steady-state alterations to the rate of membrane synthesis and breakdown and/or changes in cell density.

In addition, the ACC also behaves differently in the task state. Bipolar depression adolescents were differentiated from unipolar depression adolescents by lower neural activity in the ACC and other brain areas, including the frontal precentral cortex and temporal cortex, in both happy and fearful experiments (Diler et al., 2013). Under the NoGo condition in the GoNoGo task, ACC activity relative to HC was significantly higher only in BD—not MDD—adolescents (Diler et al., 2014). Furthermore, other brain regions, including the dorsolateral prefrontal cortex (DLPFC), were positively associated with increased reaction time in unipolar depression adolescents, whereas this relationship was not found in bipolar depression adolescents. This suggests that brain functional characteristics during the task state may also be a marker to distinguish BD from MDD.

To conclude this section, the studies reviewed here indicated that ACC plays an important role in distinguishing MDD from BD in adolescents. Belonging to the affective control network, the ACC is a key center integrating the affective neuron connections (Scotti-Muzzi et al., 2021; Li et al., 2022). Therefore, the distinctive ability of ACC in unipolar depression and bipolar depression may be explained by the fact that ACC can regulate the emotional state (Seamans and Floresco, 2022).

Taken together, imaging metrics are very promising as biomarkers to differentiate MDD from BD in adolescents. The future direction could be to combine different indicators to build models that may increase the utilization of imaging data and improve the accuracy of differentiation (Liu et al., 2022). Alternatively, big data analysis can be performed in combination with imaging databases, such as the UK Biobank dataset, to find suitable biomarkers.

#### 4.2.4. Peripheral blood biomarkers

Ideal biological biomarkers are supposed to be objective and applicable tools for the early differentiation of BD. Peripheral blood biomarkers refer to biological indicators that can be obtained from a patient's peripheral blood and used for the diagnosis, prediction, or monitoring of diseases, such as proteins, metabolites, and cytokines. In the field of psychiatric disorders, peripheral blood biomarkers are also increasingly becoming of interest to scientists because they are accessible, quantifiable, and economical.

Although not shown as clearly and centrally as cluster, considering that the detection of neuroimaging information and genetic background may have equipment and technical limitations in clinical practice, we also included stable and easily detectable peripheral blood indicators such as inflammatory cytokines and metabolic levels to improve their clinical usefulness based on general literature reading.

Many studies have reported that elevated levels of inflammation play an important role in the early onset of BD and MDD in adults (Miller et al., 2009; Lesh et al., 2018), but it is unclear whether the same changes are manifested in adolescent patients. Miklowitz et al. (2016) have explored this question in a small sample, where they examined circulating levels of inflammatory cytokines as well as spontaneous and stimulated levels of activated nuclear factor kappa B (NF-κB) in total peripheral blood in 18 adolescents with BD, 13 with MDD, and 20 without a history of psychiatric disorders. They found that adolescents with BD had significantly higher spontaneous levels of NF-κB in peripheral blood mononuclear cells, monocyte and lymphocyte populations, and higher plasma levels of interleukin-1β (IL-1β) than healthy youth, while any inflammatory cytokine levels were not significantly altered in youth with MDD compared to either the BD or HC groups. To more visually compare the presence of imbalances in pro- and anti-inflammatory cytokines in adolescent patients with mood disorders, Chen et al. (2021) included 35 first-episode adolescent patients with BD, 29 adolescent patients with MDD, and 22 age- and sex-matched healthy controls. They detected indexes of pro-inflammatory cytokines, IL-6, C-reactive protein (CRP), and anti-inflammatory cytokines IL-2, respectively. The results showed that among the three groups, patients with BD showed the highest levels of CRP, IL-6, and TNF-α, while they had the lowest levels of IL-2; that is, adolescent patients with first-episode BD had higher levels of pro-inflammatory cytokines and lower levels of anti-inflammatory cytokines. These findings are consistent with previous results in several early stages of adult samples (Goldstein et al., 2015; Chang et al., 2017; Lin et al., 2020). It suggests that although the exact mechanism of inflammation in the pathogenesis of mood disorders is not clear, BD is more likely to be related to a high pro-inflammatory state, so it may be used as a potential indicator for differential diagnosis.

In addition, clinical studies have found a higher prevalence of metabolic syndrome in adolescents with BD (Li et al., 2019), so we also focus on whether there is a difference in metabolic indexes in patients with BD and MDD. Wu et al. (2022) found that adolescent BD patients showed higher levels of fasting blood glucose, uric acid, and lactate dehydrogenase (LDH) than MDD. Blood glucose and uric acid are closely related to energy metabolism and are the essential diagnostic elements of metabolic syndrome, so this finding is consistent with clinical observation. LDH is one of the most striking findings. LDH is the main metabolic enzyme that converts pyruvate to lactic acid, and lactic acid is a direct sign of mitochondrial dysfunction (Stork and Renshaw, 2005). There is strong evidence to support the role of mitochondrial dysfunction in the pathogenesis of BD. Previous studies have also found that the content of lactic acid in the brain and cerebrospinal fluid of BD patients is significantly higher than that of healthy controls (Kuang et al., 2018); even in the first-episode adolescent BD patients, blood lactate levels were significantly higher than in healthy controls (Jeong et al., 2020). The above results suggest that LDH may be a potential peripheral biomarker relevant to the pathogenesis of BD.

In summary, available studies suggest that peripheral blood markers like inflammatory cytokines and metabolic markers may be useful objective indicators for distinguishing between BD and MDD. However, due to the limited number of original studies and small sample sizes in adolescent patients, these findings should be interpreted with caution, and more comprehensive studies are needed.

#### 4.2.5. Conclusion and future directions

**Figure 6:**
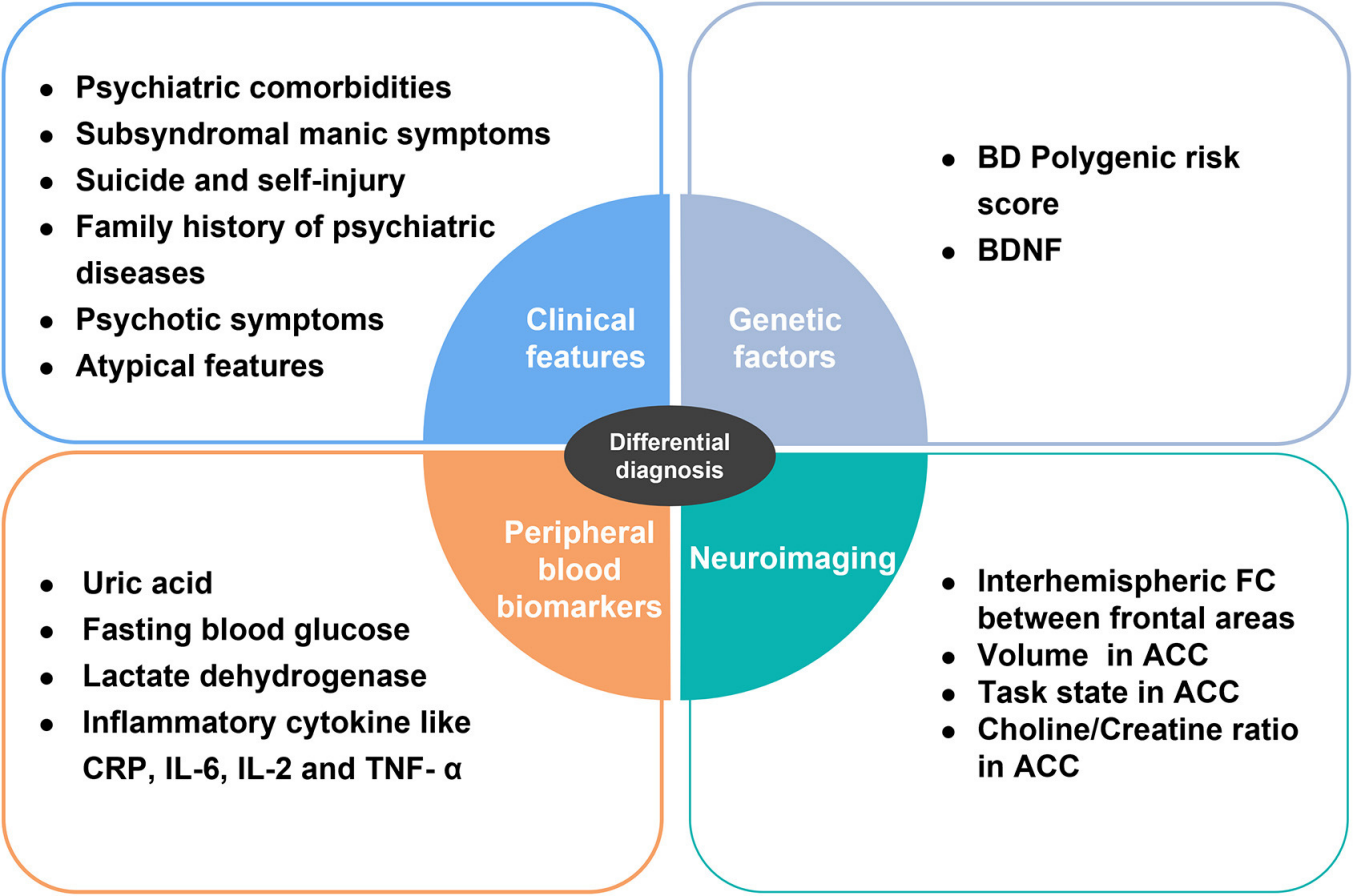
Summary of indicators differentiating BD from MDD. BD, bipolar disorder; BDNF, brain-derived neurotrophic factor; CRP, C-reactive protein; IL-6, interleukin-6; IL-2, interleukin-2; TNF-α, tumor necrosis factor alpha; FC, functional connectivity; ACC, anterior cingulate cortex.

BD and MDD in adolescents are sometimes difficult to differentiate in clinical practice due to the overlap of depressive symptoms and the resulting serious consequences, such as the chronicity of the course and increasing suicide rates. However, by visualizing the important directions provided by bibliometric analysis, we reviewed the available cross-sectional comparative literature on BD and MDD in adolescents and found that clinical features, genetic factors, neuroimaging, and peripheral blood indicators can give us some clues (Figure 6).

In general, first of all, BD is more likely to exhibit more psychiatric co-morbidity, atypical features, psychotic features, risk of self-inflicted suicide, subsyndromal manic symptoms, and a high rate of positive family history. Second, patients with BD have higher BD PRS and lower brain-derived neurotrophic factor (BDNF) gene expression levels than MDD in some cases. Third, the interhemispheric FC between the frontal areas of BD individuals and the white and gray matter of the right ACC was enhanced, and the choline/creatine ratio of the ACC was decreased. In addition, BD showed higher ACC activity in the task state. Fourth, BD has higher levels of pro-inflammatory cytokines, including CRP, IL-6, and TNF-α and lower levels of anti-inflammatory cytokines such as IL-2. In addition, BD patients have more severe metabolic disorders, reflected in elevated fasting blood glucose, uric acid, and lactate dehydrogenase.

At present, there are relatively few studies directly comparing the characteristics of BD and MDD in adolescents, and the sample size is small. Therefore, the above research results should be regarded as preliminary and need to be further confirmed. Despite this limitation, our review of the literature shows that clinical features such as more psychiatric co-morbidities and more severe subsyndromal manic symptoms can be repeated in a number of studies in different regions, suggesting that we should focus on the exploration of objective biomarkers in the next step.

Regarding the genetic aspect, in addition to the detection of mutational information, the researchers investigated the effectiveness of a range of other genetic biomarkers for differential diagnosis, including genes involved in synaptic plasticity, neurogenesis, mood control, brain aging, immune-inflammatory processes, and genes that play fundamental roles in the mitochondrial respiratory chain. Menezes et al. (2019) found that the gene expression levels of BDNF were the lowest in BD and the second lowest in MDD compared to healthy controls, and the results of this study were well replicated. This suggests that gene expression of BDNF may be one of the genetic biomarkers worthy of in-depth study. However, the shortcoming is that most of the current literature on genetic biomarkers of mood disorders focuses on adult patients. The literature aiming to use genetic biomarkers for early identification of MDD and BD in adolescents is almost non-existent. Therefore, in order to achieve identification of BD and MDD and reduce misdiagnosis rates among adolescents, future research could be explored in this way.

In the field of neuroimaging, while there have been fMRI studies that have explored the differences between BD and MDD in adolescents, these studies have not been able to dynamically assess changes in the brain over time, and electroencephalography (EEG) can fill this gap in functional MRI because it has good temporal resolution and can capture rapid changes in neuronal network dynamics (Baumgartner and Koren, 2018) and is therefore a promising diagnostic marker of disease. Indeed, in recent years, EEG analysis has been applied to patients with psychiatric disorders such as BD (Vellante et al., 2020), MDD (Damborská et al., 2019; Murphy et al., 2020), attention deficit hyperactivity disorder (ADHD) (Han et al., 2022), schizophrenia (Cao et al., 2022), and others. Different psychopathological states exhibit different changes in EEG characteristics. However, no direct comparative studies in adolescents with BD and MDD have been published, making it an area worth exploring.

Furthermore, Wu et al. (2022) explored targeted screening of peripheral blood indicators in adolescent patients with mood disorders, and found that the diagnostic model constructed using four indicators of direct bilirubin, LDH, free triiodothyronine, and CRP had a moderate identification effect in external validation. However, the coverage of targeted screening is small, and other more meaningful indicators may be overlooked. With the rapid development of high-throughput sequencing technology, molecular diagnosis has evolved from single-molecule diagnosis to encompass multi-molecule diagnosis, and even the integration of multiple omics disciplines (Wang et al., 2022). Therefore, researchers can try to use transcriptomics, proteomics, and metabolomics for deeper biomarker mining in peripheral blood in future studies.

Overall, the identification of clinical features and objective biomarkers that can be used to identify BD and MDD in adolescents is necessary and meaningful. In future studies, expanding the sample size of adolescents, broadening research directions such as EEG and multi-omics studies, and even using machine learning to construct multidimensional differential diagnostic models may have good clinical applications.

### 4.3. Limitations

The limitations of this study were as follows: First, due to constraints in personnel and resources, only the WoS database was selected for data collection. Although the WoS database is widely regarded as comprehensive and robust enough to support bibliometric analysis (Yeung et al., 2020), future research may benefit from expanding the search to other databases such as PubMed or Scopus. Second, the guiding characteristic of bibliometric research is not clear enough to show the trend and hotspot directly, so the interpretation of the results was made by the writer instead of the software.

## 5. Conclusion

Our study is the first ever to conduct bibliometric and visual analyses of differential diagnostic studies of BD and MDD in adolescents, revealing the current research status and important directions in the field. We aimed to explore the current state of global research in this area and identify hotspots within the field. The results of our research and analyses may provide valuable practical information for academic scholars and clinical practitioners.

## Data availability statement

The raw data supporting the conclusions of this article will be made available by the authors, without undue reservation.

## Author contributions

XL designed and directed the study. YZ completed the bibliometric analysis, for which KL provided guidance. RY, ZT, and YZ drafted the manuscript, while XL and LCa revised it. RY, YZ, and JL drew the figures. All authors revised and approved the final version of the manuscript.
